# Rift Valley Fever in Kedougou, Southeastern Senegal, 2012

**DOI:** 10.3201/eid2003.131174

**Published:** 2014-03

**Authors:** Abdourahmane Sow, Oumar Faye, Ousmane Faye, Diawo Diallo, Bakary D. Sadio, Scott C. Weaver, Mawlouth Diallo, Amadou A. Sall

**Affiliations:** Institut Pasteur, Dakar, Senegal (A. Sow, Ou. Faye, Om. Faye, D. Diallo, B.D. Sadio, M. Diallo, A.A. Sall);; University of Texas Medical Branch, Galveston, Texas, USA (S.C. Weaver)

**Keywords:** Rift Valley fever, Rift Valley fever virus, viruses, Kedougou, Senegal

**To the Editor:** Rift Valley fever (RVF) is an acute, febrile, viral disease caused by Rift Valley fever virus (RVFV), a phlebovirus of the family *Bunyaviridae* that is endemic to sub-Saharan Africa. RVF mortality and abortion rates among young domesticated ruminants and pregnant females are high.

In humans, clinical manifestations range from mild to severe syndromes, which can include neurologic, hemorrhagic, and hepatic features and retinitis, and which sometimes result in death (*1*). Diagnosis of RVF is challenging for clinicians because clinical manifestations are not specific (*2*). Heavy rainfall and flooding create conditions for emergence of RVF vectors (*Aedes* and *Culex* spp. mosquitoes), and dispersion of this disease into new areas is linked to migration of infected livestock, wildlife, or mosquitoes.

Since 1987, when the Diama dam was built, RVF outbreaks in Mauritania have been reported regularly (*3*). In Kedougou, southeastern Senegal, RVFV was isolated 4 times from *Ae. dalzieli* mosquitoes and once from a person with a mild case of RVF (*4*). We report results of a field investigation and laboratory findings for a human case of RVF detected by surveillance of acute febrile illnesses in Kedougou.

On October 16, 2012, a 27-year-old man (school teacher) who lived and worked in Baya village in the Kedougou region of Senegal (12°27′50″N, 12°28′6″W) visited the Kedougou military health post because of high fever, chills, headache, back pain, myalgia, and arthralgia that started on October 14. He reported regular contact with domesticated animals (cows, sheep, and goats) during farming.

A thick blood smear for the patient showed a positive result for malaria, and specific treatment was given. As part of surveillance for acute febrile illnesses, blood samples from the patient were tested for IgM against RVF, chikungunya, dengue, West Nile, yellow fever, Zika, and Crimean-Congo hemorrhagic fever viruses; and for viral RNA and virus (*5*,*6*). All test results for IgM against the 7 viruses were negative 

RVFV was isolated from newborn mice that were intracerebrally inoculated with a blood sample from the patient. Viral RNA was detected by reverse transcription PCR in serum from the patient. Phylogenetic analysis of the partial nonstructural protein gene on the small RNA segment showed that the RVFV isolate was closely related to a strain that had circulated in Mauritania in 2012 (Figure).

**Figure:**
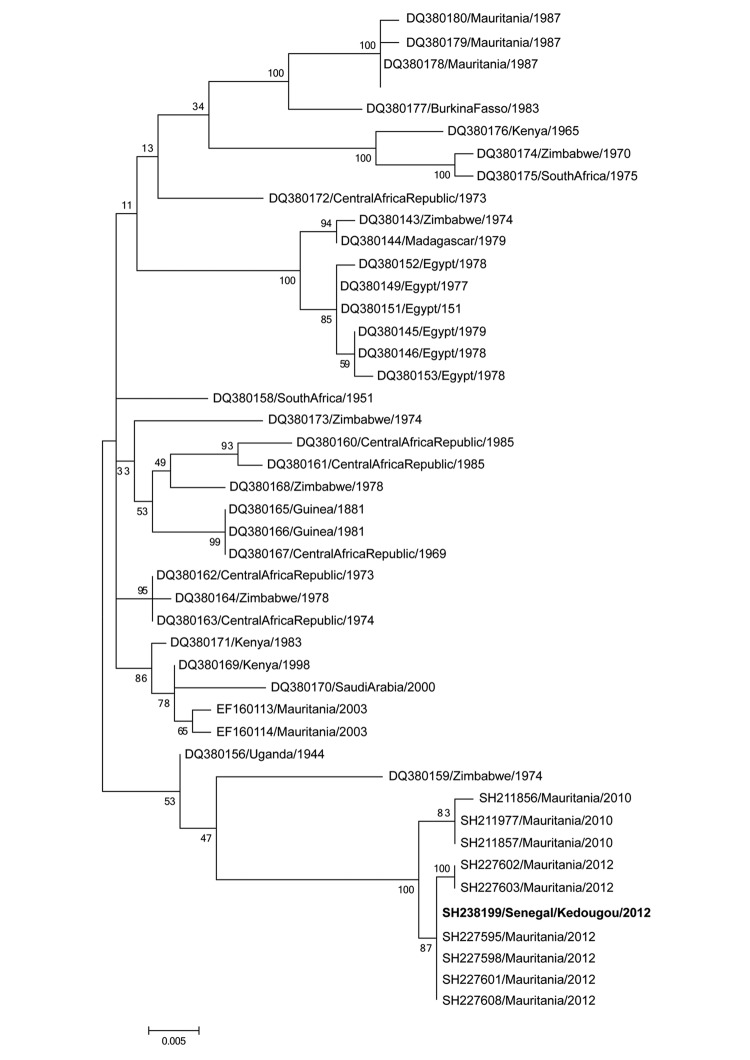
Phylogenetic tree of a 581-bp sequence of the nonstructural protein gene on the small RNA segment of Rift Valley fever viruses. **Boldface** indicates strain isolated in this study. Bootstrap values are indicated along branches. Scale bar indicates nucleotide substitutions per site.

An epidemiologic field investigation was conducted to assess the extent of RVFV circulation. During this investigation, the case-patient provided an additional blood sample. In addition, 115 contacts of the case-patient, including primary school students, friends, family members and neighbors (median age 12 years, range 6–75 years; female:male sex ratio 1.6) were also sampled and questioned to identify asymptomatic and benign cases. A total of 218 samples from patients attending the nearest health posts in Ibel and Thiokoye villages during October 2012 were also tested during surveillance of acute febrile illnesses.

All 334 samples were negative for RVFV RNA and IgM and IgG against RVFV except for samples from 3 patients, including the case-patient, which were positive for RVFV-specific IgG and malaria parasites. The 2 other patients were a 32-year-old tradesman and a 20-year-old housewife sampled during surveillance of acute febrile illnesses in Kedougou and Bandafassi, which is 30 km from Baya (Technical Appendix Figure). No RVFV RNA was detected from 519 mosquito pools sampled in the Kedougou region during October 2012, although these pools included 7 species previously found associated with RVFV and which represented 26.6 % of the pools.

The patient reported no travel outside Kedougou in the 2-year period before his illness. Because no evidence of recent RVFV circulation among humans and mosquitoes was found, we believe that the patient was infected by contact with an animal imported from Mauritania. This hypothesis is based on reports by farmers from neighboring villages (Baya, Ibel, Thiokoye, and Dondol) of the presence of ruminants imported from Mauritania in the market in Thiokoye village and of deaths and abortions among sheep and goats in their villages during October–November 2012. However, no animals were sampled during the investigation.

There is an abundance of competent vectors for RVFV in Kedougou (*4*). In addition, there are massive human migrations resulting from gold mining and regular importation of animals from RVF-endemic regions of western Africa. Thus, an integrated human and animal surveillance system should be implemented or reinforced to avoid large-scale RVF outbreaks in Kedougou.

Technical Appendix Figure. Geographic distribution of Rift Valley fever cases, southeastern Senegal, 2012.
