# Supplementary material for: Rift Valley Fever in Kedougou, Southeastern Senegal, 2012
Source: Emerg Infect Dis. 2014 Mar;20(3):504–6. doi: 10.3201/eid2003.131174 (PMC3944877; doi:10.3201/eid2003.131174)
Supplement: Technical Appendix — Figure. Geographic distribution of Rift Valley fever cases, southeastern Senegal, 2012. [file 13-1174-Techapp-s1.pdf]

Article DOI: http://dx.doi.org/10.3201/eid2003.131174

# Rift Valley Fever in Kedougou, Southeastern Senegal, 2012

## Technical Appendix

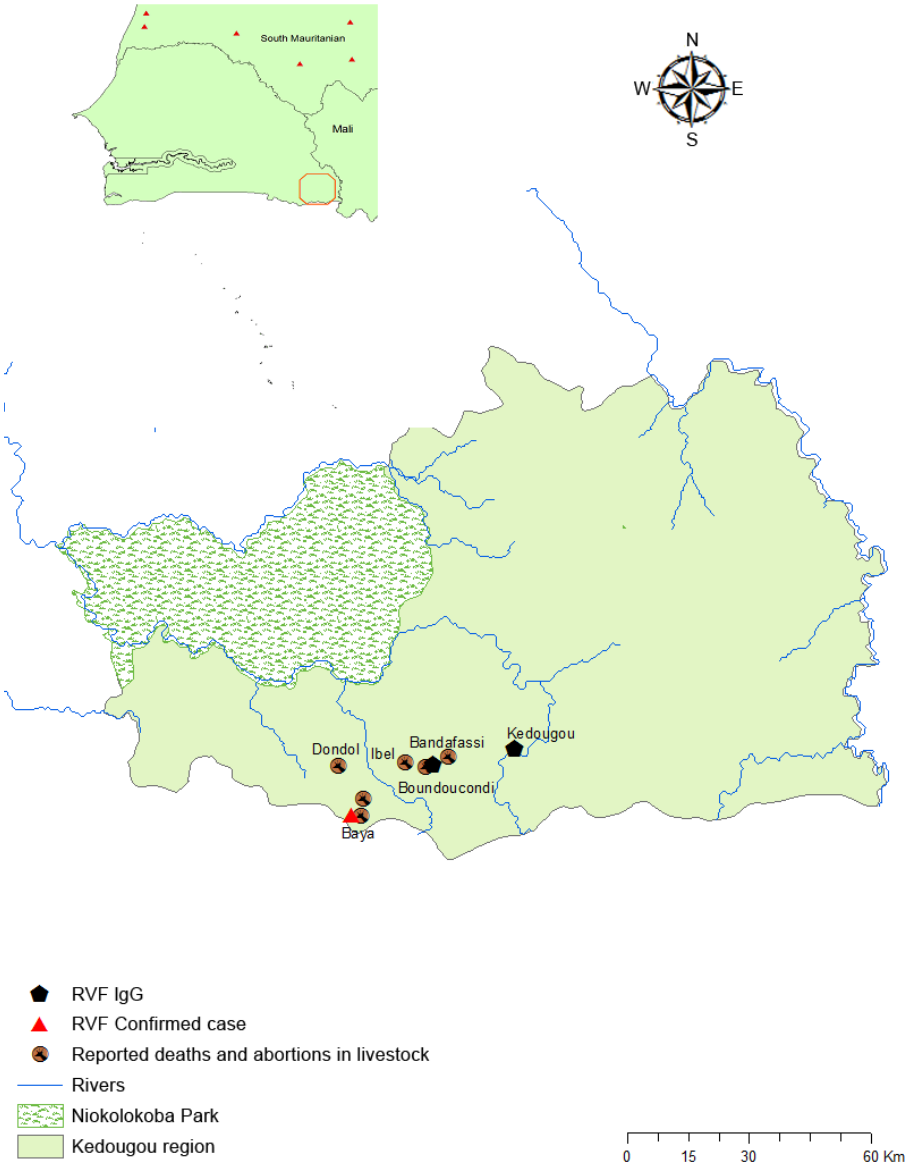


Technical Appendix Figure. Geographic distribution of Rift Valley fever cases, southeastern Senegal, 2012.
